# The Impact of Mindfulness-Based Stress Reduction (MBSR) on Psychological Outcomes and Quality of Life in Patients With Lung Cancer: A Meta-Analysis

**DOI:** 10.3389/fpsyg.2022.901247

**Published:** 2022-06-28

**Authors:** Xu Tian, Li-Juan Yi, Chen-Si-Sheng Liang, Lei Gu, Chang Peng, Gui-Hua Chen, Maria F. Jiménez-Herrera

**Affiliations:** ^1^Nursing Department, Universitat Rovira i Virgili, Tarragona, Spain; ^2^School of Nursing, Hunan Traditional Chinese Medical College, Zhuzhou, China; ^3^Medical School, Hunan Traditional Chinese Medical College, Zhuzhou, China; ^4^Sports and Arts College, Hunan Traditional Chinese Medical College, Zhuzhou, China; ^5^College of Physical Education, Hunan Traditional Chinese Medical College, Zhuzhou, China; ^6^Department of Nursing, The Second Affiliated Hospital of Chongqing Medical University, Chongqing, China

**Keywords:** lung cancer, mindfulness-based stress reduction, physical and psychological wellbeing, quality of life, meta-analysis

## Abstract

**Objective:**

The impact of the mindfulness-based stress reduction (MBSR) program on psychological outcomes and quality of life (QoL) in lung cancer patients remains unclear. This meta-analysis aimed to evaluate the effectiveness of the MBSR program on psychological states and QoL in lung cancer patients.

**Methods:**

Eligible studies published before November 2021 were systematically searched from PubMed, EMBASE, Cochrane Library, PsycINFO, China National Knowledge Infrastructure (CNKI), and Wanfang databases. The risk of bias in eligible studies was assessed using the Cochrane tool. Psychological variables and QoL were evaluated as outcomes. We used the Grading of Recommendations Assessment, Development and Evaluation (GRADE) system to grade the levels of evidence. Statistical analysis was conducted using RevMan 5.4 and STATA 14.0.

**Results:**

A total of 17 studies involving 1,680 patients were included for meta-analysis eventually. MBSR program significantly relieved cancer-related fatigue (standard mean difference [SMD], −1.26; 95% confidence interval [CI], −1.69 to −0.82; moderate evidence) and negative psychological states (SMD, −1.35; 95% CI, −1.69 to −1.02; low evidence), enhanced positive psychological states (SMD, 0.91; 95% CI, 0.56–1.27; moderate evidence), and improved quality of sleep (MD, −2.79; 95% CI, −3.03 to −2.56; high evidence). Evidence on MBSR programs' overall treatment effect for QoL revealed a trend toward statistical significance (*p* = 0.06, low evidence).

**Conclusion:**

Based on our findings, the MBSR program shows positive effects on psychological states in lung cancer patients. This approach should be recommended as a part of the rehabilitation program for lung cancer patients.

**Systematic Review Registration:**

https://archive.org/details/osf-registrations-mwvbq-v1, identifier: 10.17605/OSF.IO/MWVBQ.

## Introduction

According to the Global Cancer Statistics 2020, lung cancer ranked second for incidence and first for mortality among all types of cancers worldwide (Sung et al., 2021). Currently, several treatment modalities are available for lung cancer patients, such as surgery, chemotherapy, radiotherapy, immunotherapy, biotherapy, and complementary and alternative therapy (Gadgeel et al., 2012; Jurisevic and Bolevich, 2020; Yang and Luan, 2020); however, patients continue to suffer from serious psychological symptoms (Iyer et al., 2013; Morrison et al., 2017) because most treatment modalities simultaneously destroy both tumor and normal cells (Yang et al., 2020). Meanwhile, except for the adverse effects resulting from treatment modalities, cancer diagnosis also contributes to the development and progression of distressing symptoms (Iyer et al., 2014; Sung et al., 2017).

Patients with lung cancer have more symptom burdens than patients diagnosed with other types of cancer (Chan et al., 2009; Iyer et al., 2014; Morrison et al., 2017). Distressing symptoms can negatively affect the psychosocial wellbeing of patients with lung cancer (Yang et al., 2020; Lee, 2021). A recent study indicated that psychological stress accumulated tumor growth and increased the risk of radio-resistance associated with the activation of epithelial-mesenchymal transition by stress hormone-stimulated adrenergic receptors (Zhang et al., 2020). Moreover, several studies also demonstrated the association between high-level distressing symptoms and poor quality of life (QoL) (Möller and Sartipy, 2012; Park et al., 2016; Choi and Ryu, 2018). Fortunately, psychological interventions have been demonstrated to significantly improve the psychological wellbeing of patients with cancer (Galway et al., 2012; Huang et al., 2016; Cillessen et al., 2019).

Among the currently available psychological interventions, mindfulness-based stress reduction (MBSR), initially developed by Kabat-Zinn et al., 1998, has been widely applied in cancer settings (Lee et al., 2017; Cillessen et al., 2019). The standard MBSR program comprises an 8-week psycho-educational program and four meditative techniques, including sitting meditation, body scan, gentle Hatha yoga, and walking meditation (Kabat-Zinn et al., 1985, 1992, 1998). The exact mechanisms of the MBSR program in improving psychological wellbeing have not been fully clarified, although some studies revealed that it could affect cancer patients' neuroendocrine and immune regulation functions (Davidson et al., 2003; Robinson et al., 2003; Carlson et al., 2004; Hölzel et al., 2011). According to the previously published evidence (Kabat-Zinn et al., 1985; Kabat-Zinn and Santorelli, 2002; Kabat-Zinn, 2003), the practice of mindfulness can guide participants purposefully pay attention to the present moment and non-judgmentally monitor the unfolding of experiences moment by moment, and therefore, having a profound benefit *via* the mind-body connection.

Nevertheless, Garland et al. (2009) proposed a causal model helping to explain the mechanism of mindfulness, named as “Mindful Coping Model.” In this model, mindfulness plays a critically important role in the positive reappraisal process (Shapiro et al., 2006). Specifically, if a threat, harm, or loss exceeds one's coping capabilities, then an individual's attention may be transferred from contents to the dynamic process of consciousness by distracting stress appraisal into the model of mindfulness and then increasing individual's attentional flexibility and broadens awareness. From the vantage point of this expanded, metacognitive awareness, one can reconstrue appraisal of the given event as positive by attributing to it a new meaning, which may arise either through a conscious process of reflection or a more automatic process based on spontaneous insight. The reappraisal of the given event then triggers positive emotions to reduce stress and influences subsequent appraisal processes. According to this model and empirical evidence, destructive effects resulting from external and internal stressors (given events) may break an individual's psychosomatic balance (one's coping capabilities) and impair an individual's health status. However, mindfulness can trigger positive emotions by imitating the positive reappraisal (psychological adjustment) to restore psychosomatic balance and improve clinical outcomes.

Currently, studies have demonstrated the effectiveness and safety of the MBSR program on psychosocial wellbeing and QoL (Lee et al., 2017; Cillessen et al., 2019; Xie et al., 2020; Xunlin et al., 2020). Some studies also initially investigated the role of the MBSR program in patients diagnosed with lung diseases, such as lung cancer (van den Hurk et al., 2015; Schellekens et al., 2017) and interstitial lung diseases (Arefnasab et al., 2013; Sgalla et al., 2015). However, the benefits of the MBSR program on psychological wellbeing and QoL of lung cancer patients remain unclear because published studies reported conflicting results. More importantly, the sample size of published studies regarding lung cancer was extremely small, significantly increasing the risk of generating false results. Therefore, we performed this meta-analysis to comprehensively evaluate the effectiveness of the MBSR program on psychological outcomes and QoL of lung cancer patients.

## Methods

### Study Design

We reported all results according to the Preferred Reporting Items for Systematic Reviews and Meta-Analyses (PRISMA) statement (Page et al., 2021). We registered the protocol of this meta-analysis at the Open Science Framework (OSF) (registration number: 10.17605/OSF.IO/MWVBQ) and publicly published it in an academic journal (Tian et al., 2021). This study did not need ethical approval and patients' informed consent because it was a meta-analysis of published data.

### Information Sources

Two reviewers independently searched PubMed, EMBASE, Cochrane Library, PsycINFO, China National Knowledge Infrastructure (CNKI), and Wanfang database for relevant randomized controlled trials (RCTs) investigating the effectiveness of the MBSR program on psychological outcomes and QoL among patients with lung cancer. The literature search was limited from its inception until November 2021. The search strategy was constructed by using both the medical subject heading (MeSH) and text words, which were logically connected using Boolean operators. We also checked reference lists of previous systematic reviews with a similar topic and eligible studies to add additional studies. The consensus principle was imposed to resolve any disagreement between the two reviewers. Details of search strategies of English databases are shown in Supplementary Table 1.

### Study Selection

After removing duplicate studies, two independent reviewers conducted study selection based on the title, abstract, and full-text screening. Studies were included in the meta-analysis if they met the following criteria: (a) adult patients were cytologically or histologically diagnosed with lung cancer; (b) patients in the intervention group received both the MBSR program and usual care (UC), and patients in the control group received UC program alone, which contained at least five elements, including dietary instruction, health education, rehabilitation excise, emotional counseling, and medication instruction; (c) at least one of psychological outcomes and QoL was reported, and corresponding data were suitable for statistical analysis; (d) RCTs published in full-texts; and (e) publication language was restricted into English and Chinese because an extensive range of related research is published in English and Chinese, and no translator of other languages is available in our team. Studies were excluded if they covered at least one of the following criteria: (a) the MBSR program was designed as the part of a comprehensive strategy; (b) mixed patients were enrolled but patients with lung cancer were not separately analyzed; (c) duplicate reports of same data published by the same group; and (d) conference abstract without sufficient data.

### Data Extraction

Two reviewers independently extracted essential data from eligible studies using a predesigned standard information extraction sheet, including the first author's name, publication year, country, condition of patients, tumor stage, sample size, mean age, details of the MBSR program, outcomes, and measurements. We extracted the data at the end of the intervention or the last follow-up for statistical analysis. We contacted the corresponding author to obtain the essential data if necessary. The consensus principle was introduced to resolve the disagreement between the two reviewers.

### Risk of Bias Assessment

Two reviewers independently assessed the risk of bias in eligible studies using the Cochrane risk of bias assessment tool from seven items (Higgins et al., 2011): random sequence generation, allocation concealment, blinding of personnel and participants, blinding of outcome assessor, incomplete outcome data, selective outcome reporting, and other bias sources. Each item was rated as “low,” “unclear,” or “high” risk according to the matching level between actual information and assessment criteria. The level of overall methodological quality was judged as “high” if all items were rated as “low” risk of bias, as “low” if at least one item was rated as “high” risk of bias, and “moderate” if at least one item was rated as “unclear” risk of bias, but no item was rated as “high” risk of bias. The consensus principle was introduced to resolve the disagreement between the two reviewers.

### Statistical Analysis

Statistical analysis was conducted using Review Manager (RevMan) 5.4 (Cochrane Collaboration, Oxford, United Kingdom) and STATA 14.0 (StataCorp, Texas, USA). All outcomes were continuous variables in this meta-analysis. We, therefore, used mean difference (MD) or standard mean difference (SMD) with a 95% confidence interval (CI) to express all pooled results. We comprehensively evaluated statistical heterogeneity using the Chi-square test (Cochrane Q) and *I*^2^ statistic (Higgins and Thompson, 2002; Higgins et al., 2003). Substantial statistical heterogeneity was considered if the *p*-value was <0.1 and *I*^2^ was more than 50%. Nevertheless, we used the random-effects model to conduct a meta-analysis because variations across studies are inevitable in real settings. We also designed a series of subgroup analyses to investigate the influence of the MBSR program on different functional dimensions. We did not perform a publication bias test because the number of eligible studies for individual outcomes did not exceed 10 (Egger et al., 1997; Sterne and Egger, 2001; Page et al., 2018). Statistical significance was judged based on two-tail, and a *p*-value of <0.05 was regarded as the cutoff value of statistical significance.

### Quality of Evidence Assessment

Two independent reviewers used the Grading of Recommendations Assessment, Development and Evaluation system (Guyatt et al., 2008) to rate the level of evidence as “high,” “moderate,” “low,” or “very low.” With the GRADE system, the level of RCT was initially rated as high, and 5 factors could downgrade the level, including the risk of bias, inconsistency, indirectness, imprecision, and publication bias. Certainly, some factors could also upgrade the level of evidence, such as large effects. The consensus principle was introduced to resolve the disagreement between the two reviewers.

## Results

### Study Selection

**Figure 1:**
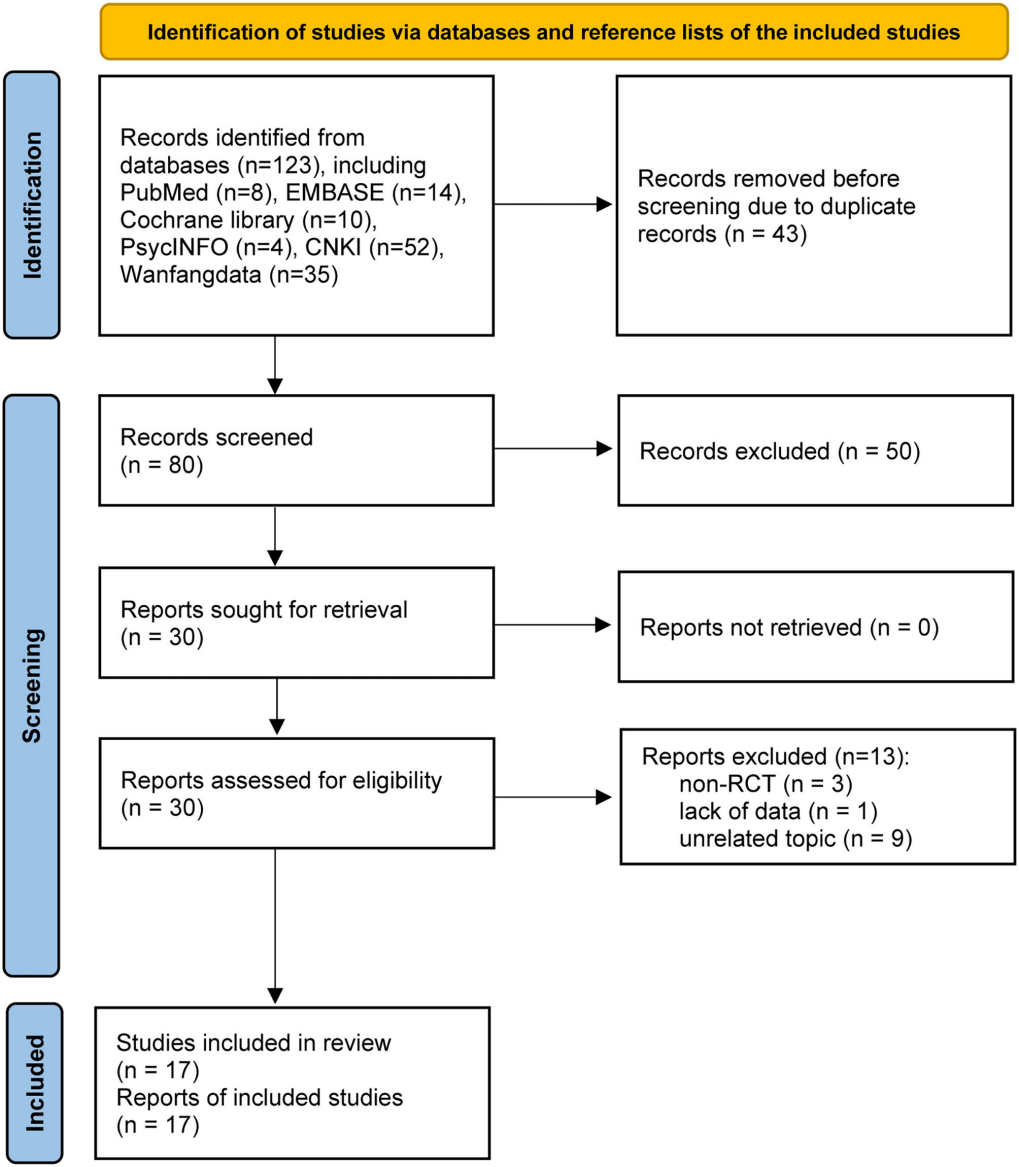
PRISMA flowchart of study retrieval and selection process. CNKI, China National Knowledge Infrastructure.

Figure 1 shows the process of study retrieval and selection. We identified 123 records from the database. Notably, 43 duplicate records were first removed. Then, 50 studies were excluded after checking their titles and abstracts. Thirty studies were further assessed for eligibility, while 13 studies were excluded due to: (a) non-RCT design (*n* = 3), (b) lack of essential data (*n* = 1), and (c) unrelated topic (*n* = 9). Finally, 17 studies were included in this meta-analysis (Ning et al., 2017; Schellekens et al., 2017; Wang et al., 2017; Guan and Zhou, 2018; Liu, 2018; Tang et al., 2018; Chen et al., 2019; Liu J. L. et al., 2019; Liu T. et al., 2019; Tian et al., 2019; Wang, 2019; Xu et al., 2019; Ding and Chu, 2020; Feng and Gong, 2020; Wu, 2020; Xi et al., 2020; You, 2020).

### Characteristics of Included Studies

All studies (Ning et al., 2017; Wang et al., 2017; Guan and Zhou, 2018; Liu, 2018; Tang et al., 2018; Chen et al., 2019; Liu J. L. et al., 2019; Liu T. et al., 2019; Tian et al., 2019; Wang, 2019; Xu et al., 2019; Ding and Chu, 2020; Feng and Gong, 2020; Wu, 2020; Xi et al., 2020; You, 2020) were conducted in China except for one study, which was conducted in the Netherlands (Schellekens et al., 2017). All studies were published between 2017 and 2020. The sample size of individual study ranged from 36 to 378, with a total number of 1,680. A total of 13 studies (Ning et al., 2017; Schellekens et al., 2017; Wang et al., 2017; Guan and Zhou, 2018; Liu, 2018; Tang et al., 2018; Chen et al., 2019; Tian et al., 2019; Xu et al., 2019; Ding and Chu, 2020; Feng and Gong, 2020; Wu, 2020; Xi et al., 2020) used standard 8-week MBSR program, but four studies used modified MBSR protocols, including 3-week program (Liu T. et al., 2019), 4-week program (Liu J. L. et al., 2019; Wang, 2019), and 6-week program (You, 2020). The remaining characteristics of eligible studies are shown in Table 1.

**Table 1:** Basic characteristics of the included studies (*n* = 17).

**References**	**Country**	**Patients**	**Stage**	**Sample for analysis**		**Mean age, years**		**Details of MBSR**	**Follow-up**	**Outcomes**	**Instrument**
				**MBSR**	**UC**	**MBSR**	**UC**				
Wang et al. (2017)	China	Lung cancer underwent surgery and chemotherapy	0, I–III	33	34	n.a.	n.a.	8-week MBSR program practice under the guidance and supervision of a qualified nurse, consisting of 8 weekly group-based 2-h sessions and once-daily 30-min self-training. Patients were supervised to daily practice after discharge by a nurse using telephone or WeChat.	8 weeks	CRF	RPFS
Liu (2018)	China	Lung cancer	n.a.	31	31	62.5	62.3	8-week MBSR program practice under the guidance and supervision of a qualified nurse, consisting of 8 weekly group-based 2-h sessions and once-daily self-training. Patients were supervised to daily practice after discharge by a nurse using telephone or WeChat.	8 weeks	CRF	RPFS
Wang (2019)	China	Lung cancer	0, I–III	45	45	45	56.02	4-week MBSR program practice under the guidance and supervision of a qualified nurse, consisting of 10-min explanation and meditation in the first week, 10-min walking meditation in the second week, 10-min breathing meditation in the third week, and 20-min experience sharing in the fourth week. Patients were supervised to daily practice after discharge by a nurse twice weekly using telephone or WeChat.	4 weeks	CRF, QoL	RPFS, EORTC QLQ-C30
Ding and Chu (2020)	China	Lung cancer underwent chemotherapy	III, IV	45	45	55.26	53.59	8-week MBSR program practice under the guidance and supervision of a qualified nurse, consisting of 8 weekly group-based 2-h sessions and once-daily self-exercise. Patients were supervised to daily train after discharge by a nurse using telephone or WeChat.	8 weeks	Anxiety, depression, CRF, self-efficacy	SAS, SDS, CFS, SUPPH
Xi et al. (2020)	China	NSCLC underwent chemotherapy	III, IV	34	34	62	62	8-week MBSR protocol training under the guidance and supervision of a nurse with qualification, consisting of 8 weekly group-based 30-min sessions. Patients were supervised to train after discharge was implemented by nurses using the telephone twice per week.	8 weeks	CRF, quality of sleep	RPFS, PSQI
Guan and Zhou (2018)	China	Lung cancer underwent chemotherapy	I–IV	23	23	54.4	51.8	8-week MBSR program practice under the guidance and supervision of a qualified nurse, consisting of 6 weekly group-based 30-min sessions. Patients were supervised to daily practice after discharge by a nurse using telephone or WeChat.	8 weeks	CRF, self-efficacy	CFS, SUPPH
Xu et al. (2019)	China	Lung cancer underwent chemotherapy	II–IV	84	84	n.a.	n.a.	8-week MBSR program practice under the guidance and supervision of a qualified nurse, consisting of 6 weekly 30–45-min self-practice at 9:00–10:00 a.m. and 17:00–18:00. Patients were supervised to practice MBSR for 30–45 min daily by a nurse using the telephone after discharge.	8 weeks	Anxiety, depression, quality of sleep	SAS, SDS, PSQI
Liu T. et al. (2019)	China	Lung cancer underwent chemotherapy	II, III	50	50	54.49	57.65	3-week MBSR program practice under the guidance and supervision of a qualified nurse, consisting of 3 weekly group-based 30–40-min sessions including a 15-min explanation from a trainer and 20–30 min of training. Patients were supervised to practice MBSR for 30–45 min daily by a nurse using the telephone after discharge.	12 weeks	Self-efficacy, mindfulness	SUPPH, MAAS
Liu J. L. et al. (2019)	China	Lung cancer underwent chemotherapy	n.a.	44	44	56	55	4-week MBSR program under the guidance and supervision of a qualified nurse, consisting of 4 weekly group-based 2-h sessions and 30-min self-practice daily.	10 weeks	Anxiety, depression, QoL	SAS, SDS, EORTC QLQ-C30
Ning et al. (2017)	China	Lung cancer	n.a.	18	18	39.81	40.76	Standard 8-week MBSR program, which was accessed from www.iepsy.com, under the guidance and supervision of a qualified nurse.	8 weeks	Anxiety, depression	SAS, SDS
Tang et al. (2018)	China	Lung cancer underwent surgery and chemotherapy	I–III	36	36	53.22	50.55	8-week MBSR program under the guidance and supervision of a qualified nurse, consisting of 8 weekly group-based 2-h sessions including a 30-min explanation from a trainer, 30-min practice, 30-min question, and 30-min experience-sharing. Patients were supervised to practice MBSR daily by a nurse using the telephone after discharge.	8 weeks	CRF	RPFS
Tian et al. (2019)	China	Lung cancer underwent concurrent chemoradiotherapy	n.a.	46	46	53.51	54.12	8-week MBSR program, consisting of 8 weekly group-based 30–40-min sessions and self-practice daily. Patients were supervised to practice MBSR daily by a nurse using the telephone after discharge.	8 weeks	Psychological distress, anxiety, depression, the activity of daily living	DT, SAS, SDS, ADL
Wu (2020)	China	Lung cancer underwent chemotherapy	0, I–III	57	57	67.49	67.51	8-week MBSR program training under the guidance and supervision of a qualified nurse, consisting of 8 weekly group-based 30-min sessions and self-practice every day.	8 weeks	CRF, anxiety, depression, QoL	RPFS, HAMA, HAMD, EORTC QLQ-C30
You (2020)	China	Early lung cancer underwent surgery	n.a.	189	189	57.73	58.43	6-week MBSR program under the guidance and supervision of a nurse with qualification, consisting of 6 weekly group-based 2-h sessions, including 30-min explanation, 60-min self-practice, and 30-min experience-sharing.	6 weeks	Psychological distress, anxiety, depression, quality of sleep, performance status	DT, HAMA, HAMD, PSQI, KPS
Schellekens et al. (2017)	Netherlands	Lung cancer	I–IV	21	18	60.6	57	8-week MBSR program, consisting of 1 weekly 2.5-h group training, a silent day between sessions 6 and 7, and home practice assignments of about 45 min, 6 days per week.	3 months	Anxiety, depression, QoL, mindfulness	HADS, EORTC QLQ-C30, FFMQ
Chen et al. (2019)	China	NSCLC underwent chemotherapy	n.a.	31	32	57.83	59.11	8-week MBSR program under the guidance and supervision of a qualified nurse, consisting of 1 weekly 2-h group training and self-practice daily. Patients were supervised to daily practice MBSR by a nurse using the telephone after discharge.	8 weeks	CRF, anxiety, depression	CFS, SAS, SDS
Feng and Gong (2020)	China	Lung cancer underwent chemotherapy	n.a.	54	53	57.69	57.34	8-week MBSR program under the guidance and supervision of a qualified nurse, consisting of 1 weekly 2-h group practice at 9:00–10:00 a.m. or 17:00–18:00 and 30-min self-practice at home.	8 weeks	CRF, self-efficacy, quality of sleep	RPFS, SUPPH, PSQI

*NSCLC, non-small-cell lung cancer; CRF, cancer-related fatigue; QoL, quality of life; R-PFS, Revised Piper Fatigue Scale; CFS, Cancer Fatigue Scale; SAS, Self-rating Anxiety Scale; SDS, Self-rating Depression Scale; HAMA, Hamilton Anxiety Scale; HAMD, Hamilton Depression Scale; HADS, Hospital Anxiety and Depression Scale; DT, distress thermometer; SUPPH, strategies used by people to promote health; MAAS, Mindful Attention Awareness Scale; ADL, Activity of Daily Living Scale; PSQI, Pittsburgh sleep quality index; EORTC QLQ-C30, the European Organization for Research and Treatment of Cancer Quality of Life Questionnaire; FFMQ, Five Facet Mindfulness Questionnaire; KPS, Karnofsky performance status; MBSR, mindfulness-based stress reduction; US, usual care; n.a., not applicable*.

### Risk of Bias

The risk of bias assessment of included studies is displayed in Supplementary Figure 1. Overall, more than half of the studies (52.94%) (Ning et al., 2017; Schellekens et al., 2017; Wang et al., 2017; Chen et al., 2019; Liu J. L. et al., 2019; Tian et al., 2019; Feng and Gong, 2020; Wu, 2020; You, 2020) were judged as “low” risk of bias due to the attrition bias. Generally, the majority of studies (Ning et al., 2017; Wang et al., 2017; Guan and Zhou, 2018; Liu, 2018; Tang et al., 2018; Chen et al., 2019; Liu J. L. et al., 2019; Liu T. et al., 2019; Tian et al., 2019; Wang, 2019; Xu et al., 2019; Ding and Chu, 2020; Feng and Gong, 2020; Wu, 2020; Xi et al., 2020; You, 2020) did not report details of allocation concealment and blinding of personnel, participants, and outcome assessors.

### Cancer-Related Fatigue

**Figure 2:**
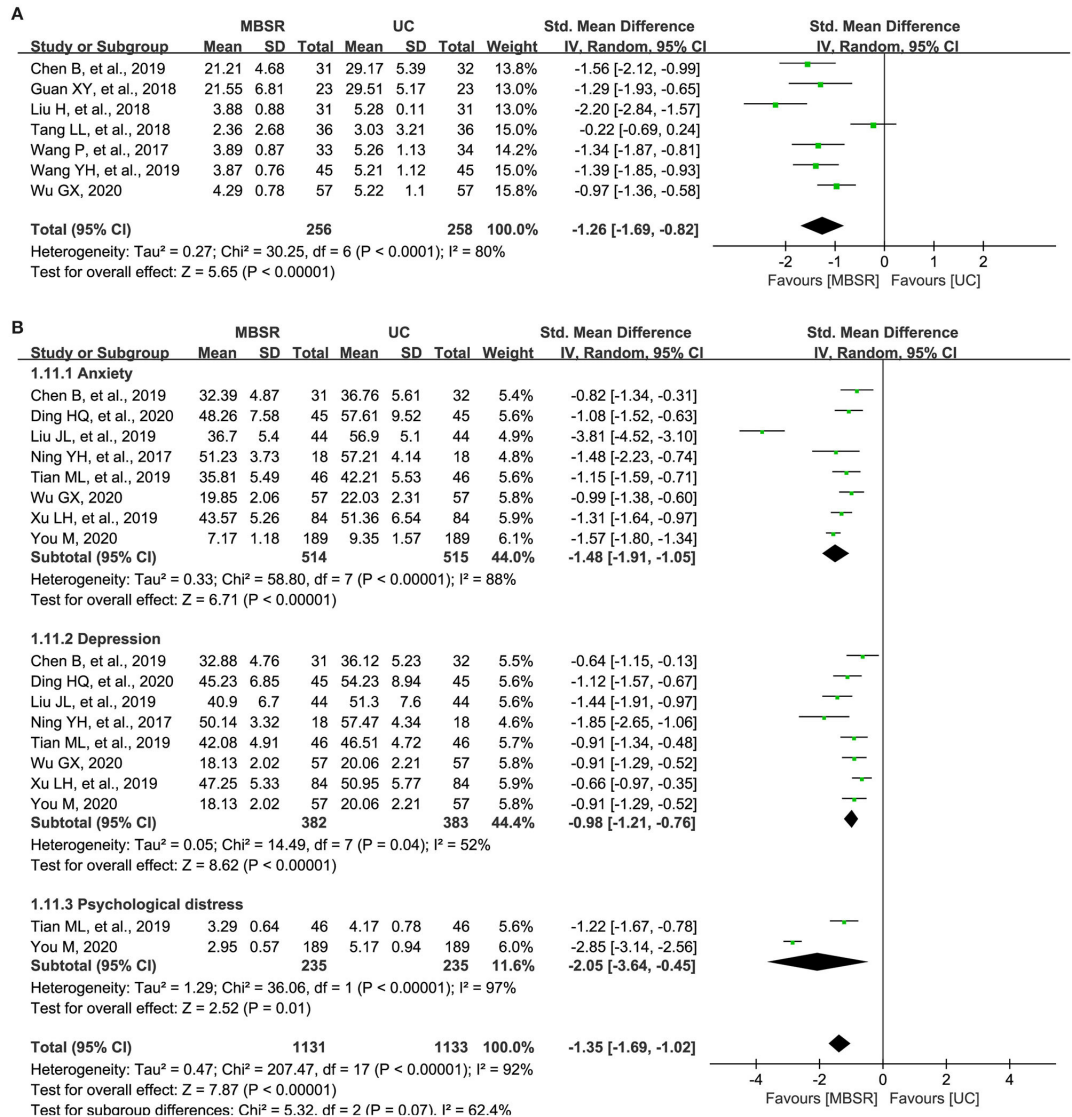
Forest plot of comparative effectiveness between mindfulness-based stress reduction (MBSR) program and UC in terms of cancer-related fatigue **(A)** and negative psychological states **(B)**. MBSR, mindfulness-based stress reduction; UC, usual care; R-PFS, Revised Piper Fatigue Scale; CFS, Cancer Fatigue Scale; SAS, Self-rating Anxiety Scale; SDS, Self-rating Depression Scale; HAMA, Hamilton Anxiety Scale; HAMD, Hamilton Depression Scale; SD, standard deviation.

A total of seven studies reported the overall level of cancer-related fatigue (Wang et al., 2017; Guan and Zhou, 2018; Liu, 2018; Tang et al., 2018; Chen et al., 2019; Wang, 2019; Wu, 2020); however, five (Wang et al., 2017; Liu, 2018; Tang et al., 2018; Wang, 2019; Wu, 2020) and three studies (Guan and Zhou, 2018; Chen et al., 2019) used the Revised Piper Fatigue Scale (R-PFS) and Cancer Fatigue Scale (CFS) to measure this outcome, respectively. Therefore, SMD was used to express the pooled results. Meta-analysis revealed a significant improvement in patients receiving MBSR program (514 patients; *I*^2^ = 80%; SMD, −1.26; 95% CI: −1.69 to −0.82; *p* <0.001; Figure 2A), which was supported by moderate evidence (Table 2).

**Table 2:** The level of evidence based on the Grading of Recommendations Assessment, Development and Evaluation (GRADE) system.

**Certainty assessment**							**No. of patients**		**Effect**	**Certainty**	**Importance**
**No. of studies**	**Study design**	**Risk of bias**	**Inconsistency**	**Indirectness**	**Imprecision**	**Other considerations**	**MBSR**	**UC**	**Absolute (95% CI)**		
**Cancer-related fatigue**											
5	Randomized trials	Serious^a^	Not serious	Not serious	Not serious	None	256	258	SMD **−1.26 lower** (−1.69 lower to −0.82 lower)	⊕⊕⊕○ Moderate	CRITICAL
**Negative psychological status**											
8	Randomized trials	Serious^b^	Not serious	Not serious	Serious^c^	Strong association	514	515	SMD **−1.35 lower** (−1.69 lower to −1.02 lower)	⊕⊕○○ Low	IMPORTANT
**Positive psychological status**											
4	Randomized trials	Not serious	Not serious	Not serious	Very serious^c^	None	148	144	SMD **0.91 SD higher** (0.56 higher to 1.27 higher)	⊕⊕⊕○ Moderate	IMPORTANT
**Quality of sleep**											
2	Randomized trials	Serious^b^	Not serious	Not serious	Not serious	Strong association	273	273	MD **2.79 lower** (3.03 lower to 2.56 lower)	⊕⊕⊕⊕ High	IMPORTANT
**Quality of life**											
3	Randomized trials	Serious^d^	Not serious	Not serious	Serious^c^	None	122	119	MD **9.55 lower** (0.47 lower to 19.58 higher)	⊕⊕○○ Low	IMPORTANT

*CI, confidence interval; MBSR, mindfulness-based stress reduction; MD, mean difference; US, usual care; SMD, standardized mean difference*.

a *Two eligible studies were judged to be at high risk of bias*.

b *One eligible study was judged to be at high risk of bias*.

c *Inadequate sample size was accumulated*.

d *Most eligible studies were judged to be at high risk of bias*.

### Negative Psychological Status

A total of eight studies (Ning et al., 2017; Chen et al., 2019; Liu J. L. et al., 2019; Tian et al., 2019; Xu et al., 2019; Ding and Chu, 2020; Wu, 2020; You, 2020) reported the changes of negative psychological states, including anxiety, depression, and psychological distress. It is noted that the level of anxiety was measured by using the Self-rating Anxiety Scale (SAS) and Hamilton Anxiety Scale (HAMA), and the level of depression was measured by using the Self-rating Depression Scale (SDS) and Hamilton Depression Scale (HAMD), and the level of psychological distress was measured using Distress Thermometer (DT). Therefore, SMD was selected as the measurement to express the pooled result of negative psychological states. Meta-analysis suggested that patients receiving the MBSR program had a significantly lower level of negative psychological outcomes compared with patients receiving UC alone (1,029 patients; *I*^2^ = 92%; SMD, −1.35; 95% CI, −1.69 to −1.02; *p* <0.001; Figure 2B), which was only supported by low evidence (Table 2). It is noted that the level of anxiety (1,029 patients; *I*^2^ = 88%; SMD, −1.48; 95% CI, −1.91 to −1.05; *p* <0.001), depression (765 patients; *I*^2^ = 52%; SMD, −0.98; 95% CI, −1.21 to −0.76; *p* <0.001), and psychological distress (470 patients; *I*^2^ = 97%; SMD, −2.05; 95% CI, −3.64 to −0.45; *p* = 0.01) were all significantly lower in the MBSR group.

### Positive Psychological Status

**Figure 3:**
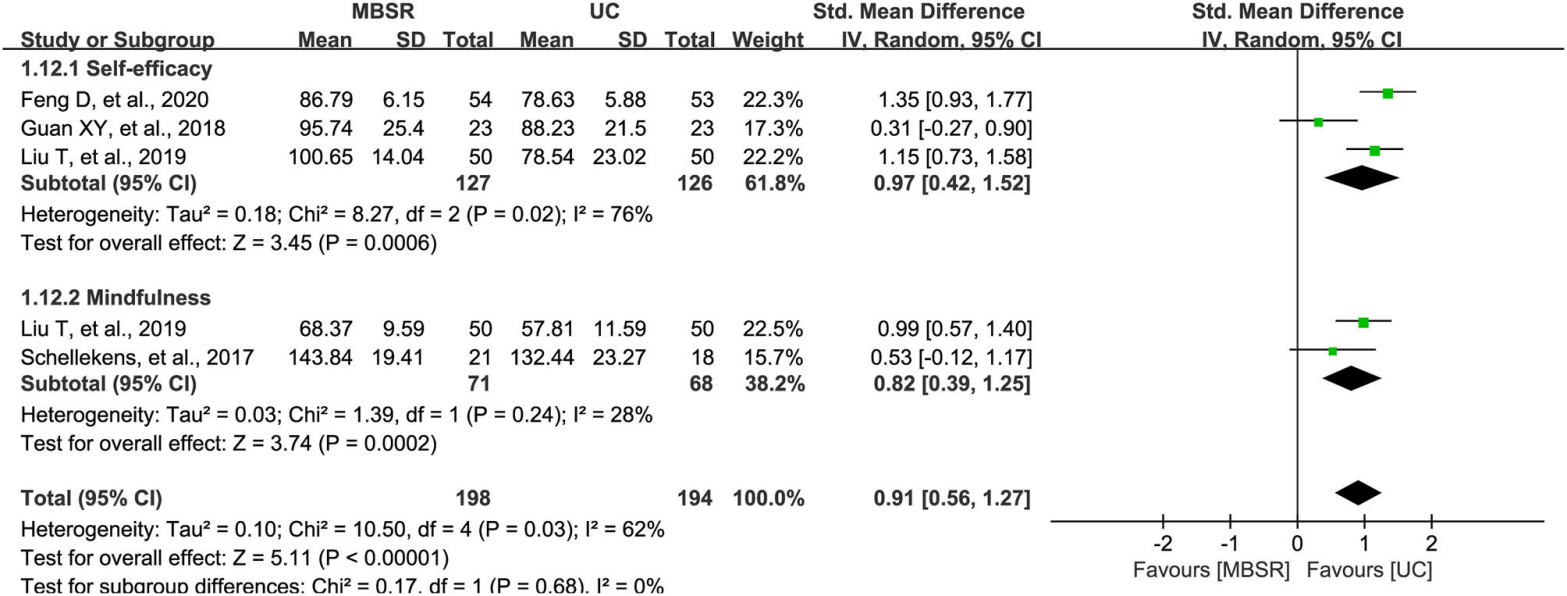
Forest plot of comparative effectiveness between the MBSR program and UC in terms of positive psychological status. MBSR, mindfulness-based stress reduction; UC, usual care; SUPPH, strategies used by people to promote health.

Among the 17 included studies, four studies reported changes in positive psychological states, including self-efficacy and mindfulness. We selected SMD to express the pooled result because self-efficacy and mindfulness were combined as an individual outcome. For self-efficacy, the “strategies used by people to promote health (SUPPH)” was used as the measurement; however, the level of mindfulness was measured by using the Mindful Attention Awareness Scale (MASS) and Five Facet Mindfulness Questionnaire (FFMQ). Meta-analysis suggested that the MBSR program significantly improved the positive psychological states (292 patients; *I*^2^ = 62%; SMD, 0.91; 95% CI, 0.56–1.27; *p* <0.001; Figure 3), which was only supported by moderate evidence (Table 2). It is noted that the level of self-efficacy (253 patients; *I*^2^ = 76%; SMD, 0.97; 95% CI, 0.42–1.52; *p* <0.001) and mindfulness (139 patients; *I*^2^ = 28%; SMD, 0.82; 95% CI, 0.39–1.25; *p* <0.001) were all significantly improved in the MBSR group.

### Quality of Sleep

**Figure 4:**
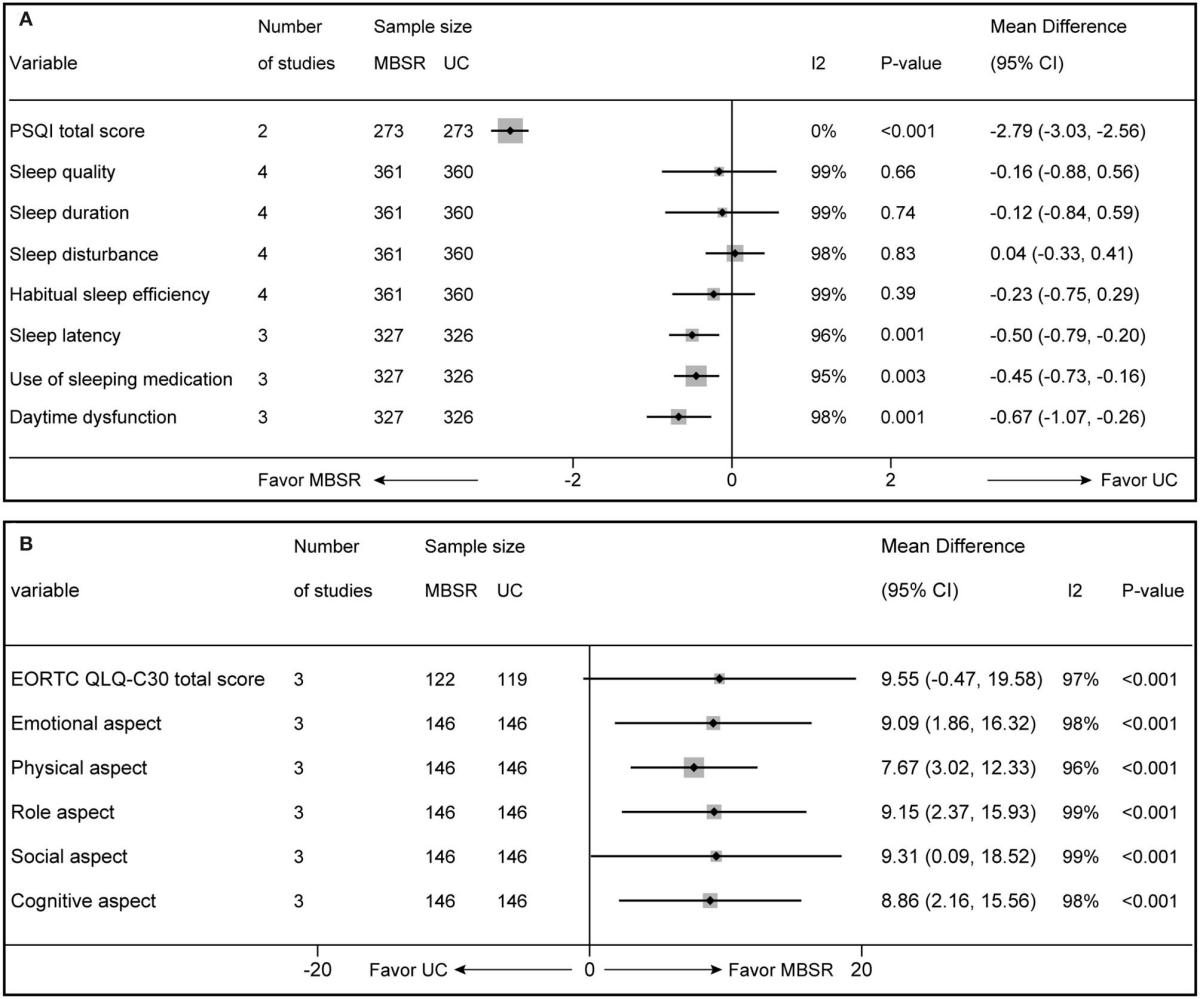
Forest plot of comparative effectiveness between MBSR program and UC in terms of quality of sleep **(A)** and QoL **(B)**. MBSR, mindfulness-based stress reduction; UC, usual care; PSQI, Pittsburgh sleep quality index; EORTC QLQ-C30, the European Organization for Research and Treatment of Cancer Quality of Life Questionnaire.

Four studies (Xu et al., 2019; Feng and Gong, 2020; Xi et al., 2020; You, 2020) reported the quality of sleep, but the total score of the Pittsburgh sleep quality index (PSQI) was available in two studies (Xu et al., 2019; You, 2020). Meta-analysis suggested that the MBSR program significantly improved the quality of sleep compared with UC alone (546 patients; *I*^2^ = 0%; MD, −2.79; 95% CI, −3.03 to −2.56; *p* <0.001; Figure 4A), which was supported by high evidence (Table 2). Subgroup analysis was conducted to investigate the impact of the MBSR program on different dimensions, including sleep quality, sleep duration, sleep disturbance, habitual sleep efficiency, sleep latency, use of sleeping medication, and daytime dysfunction, and pooled results revealed that the MBSR program only significantly influences sleep latency, use of sleeping medication, and daytime dysfunction (Figure 4A).

### Quality of Life

Four studies (Schellekens et al., 2017; Liu J. L. et al., 2019; Wang, 2019; Wu, 2020) reported QoL, but only 3 (Schellekens et al., 2017; Liu J. L. et al., 2019; Wu, 2020) provided total EORTC QLQ-C30 score. Meta-analysis on MBSR programs' overall treatment effect for QoL revealed a trend toward statistical significance (241 patients; *I*^2^ = 97%; MD, 9.55; 95% CI, −0.47–19.58; *p* = 0.06; Figure 4B), which was only supported by low evidence (Table 2). However, subgroup analysis revealed that MBSR had considerable influence on all dimensions, including emotional, physical, role, social, and cognitive aspects (Figure 4B).

## Discussion

Lung cancer remains the leading type of cancer worldwide, accounting for ~11.4% of new cancer cases and 18.0% of cancer-related deaths in 2020 (Sung et al., 2021). Patients with lung cancer suffer from a significant psychological symptom burden resulting from the destructive effects of anticancer treatment and cancer diagnosis (Iyer et al., 2014; Morrison et al., 2017), which greatly impair an individual's psychological wellbeing and reduce QoL (Iyer et al., 2013; Morrison et al., 2017). Psychological interventions have a positive impact on psychological outcomes among patients with cancer, and as a common type of psychological intervention, MBSR has also been extensively demonstrated to have a positive role in improving psychological outcomes among patients with cancer (van den Hurk et al., 2015; Lee et al., 2017; Xie et al., 2020). However, the role of the MBSR program in the treatment of patients with lung cancer has not yet been fully investigated.

In this meta-analysis, we obtained a comprehensive summary of studies investigating the effectiveness of the MBSR program on psychological outcomes (negative vs. positive aspects), quality of sleep, and QoL in lung cancer patients. Findings of this meta-analysis suggest that the MBSR program significantly relieves cancer-related fatigue, improves negative psychological states, including anxiety, depression, and psychological distress, enhances positive psychological states, including self-efficacy and mindfulness, and improves the quality of sleep. Unfortunately, meta-analysis does not reveal a statistical difference in QoL between the MBSR program and UC alone. However, the MBSR program tends to have a beneficial influence on QoL. Meanwhile, subgroup analysis suggests that the MBSR program significantly improved all dimensions of QoL compared with UC alone.

Till present, only one meta-analysis (Xie et al., 2020) investigated the effects of the MBSR program on cancer-related fatigue of patients with lung cancer based on subgroup analysis. In this meta-analysis, 3 eligible studies involving 185 patients with lung cancer were included to evaluate the effects of the MBSR program on cancer-related fatigue, and the result suggested that the MBSR program was significantly associated with a decreased level of cancer-related fatigue compared with UC alone (SMD, −0.95; 95% CI, −1.74 to −0.15; *p* = 0.02). Although the previous meta-analysis reported a consistent result with our meta-analysis in terms of cancer-related fatigue, our meta-analysis has more strengths than the previous meta-analysis. First and foremost, apart from cancer-related fatigue, the current meta-analysis also evaluated psychological variables, quality of sleep, and QoL. Moreover, we categorized psychological status into negative and positive aspects, which let us fully know that MBSR improves an individual's health status by simultaneously improving positive psychological states (i.e., the level of mindfulness and self-efficacy) and relieving negative psychological states (i.e., the level of anxiety, depression, and psychological distress). Second, this meta-analysis also used the GRADE system to rate the levels of evidence, which greatly facilitated clinical decision-making. Third, more eligible studies were included in our meta-analysis to greatly increase the statistical power. Although most included studies reported beneficial results to the MBSR program, the insufficient sample size greatly decreased the statistical power of the findings. Specifically, the sample size of individual studies ranged from 36 to 378, and more than 94% of eligible studies involved a sample size of <200. As stated previously, a total of 1,680 patients were accumulated to significantly increase the statistical power of this meta-analysis. Therefore, more reliable and robust results could be generated from the current meta-analysis compared with previous individual studies. Fourth, distress has been regarded as the sixth vital sign in the care of cancer persons (Stapleton et al., 2017; Fitch et al., 2018); however, the current meta-analysis found that limited studies evaluated the effect of the MBSR program on psychological distress of patients with lung cancer, which provides valuable implications for designing the future study. More importantly, this meta-analysis revealed that most studies were dedicated to evaluating the effectiveness of the MBSR program in physical and psychological wellbeing, but few studies tried to elucidate the potential mechanisms of the MBSR program in improving physical and psychological wellbeing. Therefore, future studies should be designed to clarify the possible mechanisms of the MBSR on different clinical outcomes from multiple perspectives.

**Figure 5:**
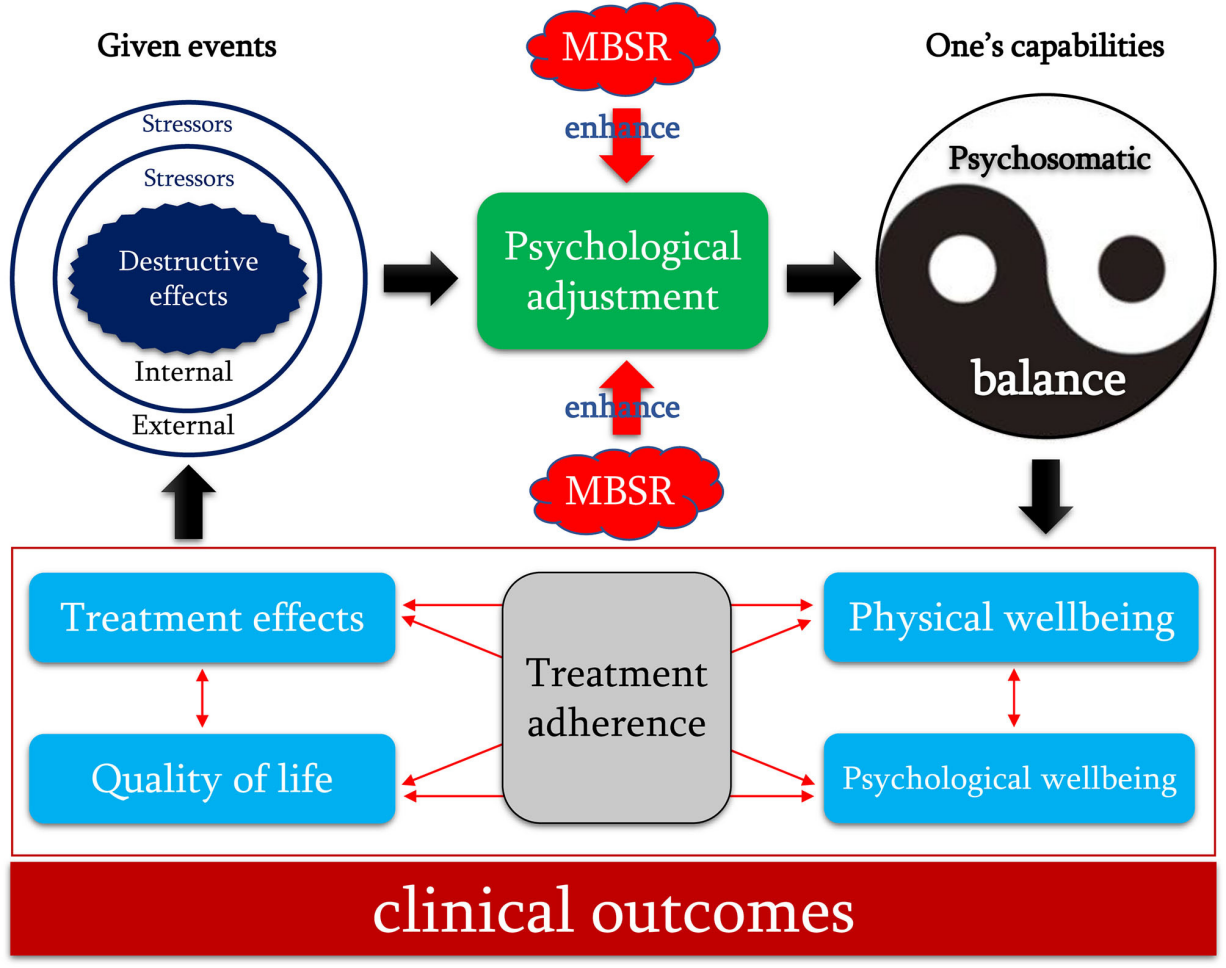
The hypothetical causal pathway of the MSBR program for improving psychological outcomes and QoL of lung cancer patients. MBSR, mindfulness-based stress reduction; QoL, quality of life. In this hypothetical causal pathway, the black unidirectional arrow indicated the causal relationship between two elements, and the red bidirectional arrow indicated the interrelationship of two elements. Destructive effects of stressors break a patient's psychosomatic balance by initiating negative psychological adjustment and then harming clinical outcomes. In contrast, implementation of the MBSR program may enhance positive psychological adjustment by triggering positive psychological sources (e.g., self-efficacy) to gradually restore the patient's psychosomatic balance and then improve clinical outcomes.

Generally, the psychosomatic balance may be a moderator of psychological well-being adjustment in patients with cancer (Bãrbuş et al., 2017). As a result, people may suffer from significant symptom burden, such as cancer-related fatigue (Besika et al., 2021) when internal (e.g., confirmation of the diagnosis of cancer) or external (e.g., anticancer treatment) stressors destructed psychosomatic balance. Then, people may suffer from a great reduction in psychological wellbeing and QoL (Zhang et al., 2019). According to the Mindful Coping Model (Garland et al., 2009), it is not surprising to the benefits of the MBSR program on psychological wellbeing, quality of sleep, and QoL. Studies have revealed that mindfulness-based interventions have a positive impact on symptom burden and psychological outcomes in patients with cancer (Rouleau et al., 2015). Specifically speaking, when patients suffer from destructive effects resulting from both external and internal stressors, the MBSR program initiates psychological adjustment to trigger positive psychological sources (positive reappraisal) (Jeffers et al., 2019), which may greatly dilute the destructive effects of stressors (Galante et al., 2021) and then restore patient's psychosomatic balance. As a result, patients' health outcomes would be significantly improved. As an example, studies have suggested that the MBSR program greatly decreased patients' symptom burden (physical wellbeing) and improved patients' psychological wellbeing and QoL (Zimmaro et al., 2020; Kim et al., 2021). Moreover, empirical studies suggested that the MBSR program also improved patients' treatment adherence and then enhanced the anticancer treatment effects, as well as improved physical status, psychological wellbeing, and QoL (Cillessen et al., 2020). According to the “Mindful Coping Model” and findings from empirical studies, we, therefore, proposed the hypothetical causal pathway that argues for the role of the MBSR program in regulating lung cancer patients' psychological wellbeing (Figure 5). It is noted that these potential influence pathways of the MBSR program on the adjustment in psychological status are speculated from previously published studies. Therefore, definitive mechanisms of the MBSR program from different aspects should be further clarified in patients with lung cancer because this meta-analysis has revealed the effectiveness of the MBSR program on psychological outcomes.

To our knowledge, this is the first study that investigated the effectiveness of the MBSR program on psychological outcomes, quality of sleep, and QoL in patients with lung cancer with a meta-analysis technique, and several promising findings provide a valuable reference for developing the socio-psychological rehabilitation program of patients with lung cancer. However, we must acknowledge that several limitations may impair the robustness and reliability of our findings. First, we systematically searched several electronic databases, including PubMed, EMBASE, PsycINFO, Cochrane Library, CNKI, and Wanfang, to identify relevant studies; however, some potentially eligible studies may be missed from our literature retrieval because other databases, such as Web of Science and SCOPUS, were not searched. Second, the substantial variations in the intensity, frequency, and duration of the MBSR program across eligible studies may introduce heterogeneity, which also may reduce the robustness of the pooled results. However, we utilized the random-effects model to conservatively estimate the effects of the MBSR program on psychological outcomes, quality of sleep, and QoL. Nevertheless, we still believe that it is essential to apply for a standard MBSR program in clinical practice to ensure interventional efficacy. Third, details of UC across studies were different, which also a potential source of introducing statistical heterogeneity. However, we defined five essential elements of UC protocol to ensure the similarity of various strategies, including dietary instruction, health education, rehabilitation excise, emotional counseling, and medication instruction. Fourth, the baseline status of patients with lung cancer was also different from one to another; however, subgroup analysis was not imposed due to limited data. Fifth, we could not quantitatively evaluate the impact of the MBSR program on the physical status because only one study reported this outcome. Sixth, most results of this meta-analysis were only supported by low to moderate evidence except for the quality of sleep. Therefore, attention should be specially paid to the interpretation of our findings. Seventh, we used the first version of the Cochrane risk of bias assessment tool for methodological quality assessment in this meta-analysis. However, a second version is being published, which should be cited in the future study.

## Conclusion

The results of this meta-analysis suggest that the MBSR program is an effective psychological approach to relieve cancer-related fatigue, and negative emotional states, including anxiety and depression, psychological stress, and improving self-efficacy, mindfulness, and quality of sleep among patients with lung cancer. Therefore, it is worthy of being recommended to patients with lung cancer as part of their rehabilitation protocol. Certainly, future studies are warranted to further investigate the effects of the MBSR program on psychological distress, level of mindfulness, and QoL because these three outcomes are only supported by low evidence. Moreover, the impact of the MBSR program on the psychological states should also be investigated in future studies because it was evaluated by only one study.

## Data Availability Statement

The original contributions presented in the study are included in the article/Supplementary Material, further inquiries can be directed to the corresponding authors.

## Author Contributions

Conception and design: XT and MJ-H. Administrative support: XT, G-HC, and MJ-H. Provision of study materials or patients: XT and L-JY. Collection and assembly of data: XT, L-JY, and C-S-SL. Data analysis and interpretation: XT, L-JY, LG, CP, and G-HC. Manuscript writing and final approval of manuscript: All authors. All authors contributed to the article and approved the submitted version.

## Funding

This study was supported by the Chongqing Natural Science Foundation (project number: cstc2020jcyj-msxmX0212) and the Medical Research Project, which was jointly approved by the Chongqing Science and Technology Bureau and Health Commission of Chongqing Municipal City (project numbers: 2022MSXM067 and 2019MSXM058).

## Conflict of Interest

The authors declare that the research was conducted in the absence of any commercial or financial relationships that could be construed as a potential conflict of interest.

## Publisher's Note

All claims expressed in this article are solely those of the authors and do not necessarily represent those of their affiliated organizations, or those of the publisher, the editors and the reviewers. Any product that may be evaluated in this article, or claim that may be made by its manufacturer, is not guaranteed or endorsed by the publisher.

## Supplementary Material

Supplementary Figure 1 Risk of bias of each eligible study. Green (+), yellow (?), and red (–) circle represents “low,” “unclear,” and “high” risk of bias, respectively.

Supplementary Table 1 Search strategies of targeted English databases.
