# Supplementary figures and images for: The Impact of Mindfulness-Based Stress Reduction (MBSR) on Psychological Outcomes and Quality of Life in Patients With Lung Cancer: A Meta-Analysis
Source: Front Psychol. 2022 Jun 28;13:901247. doi: 10.3389/fpsyg.2022.901247 (PMC9274275; doi:10.3389/fpsyg.2022.901247)

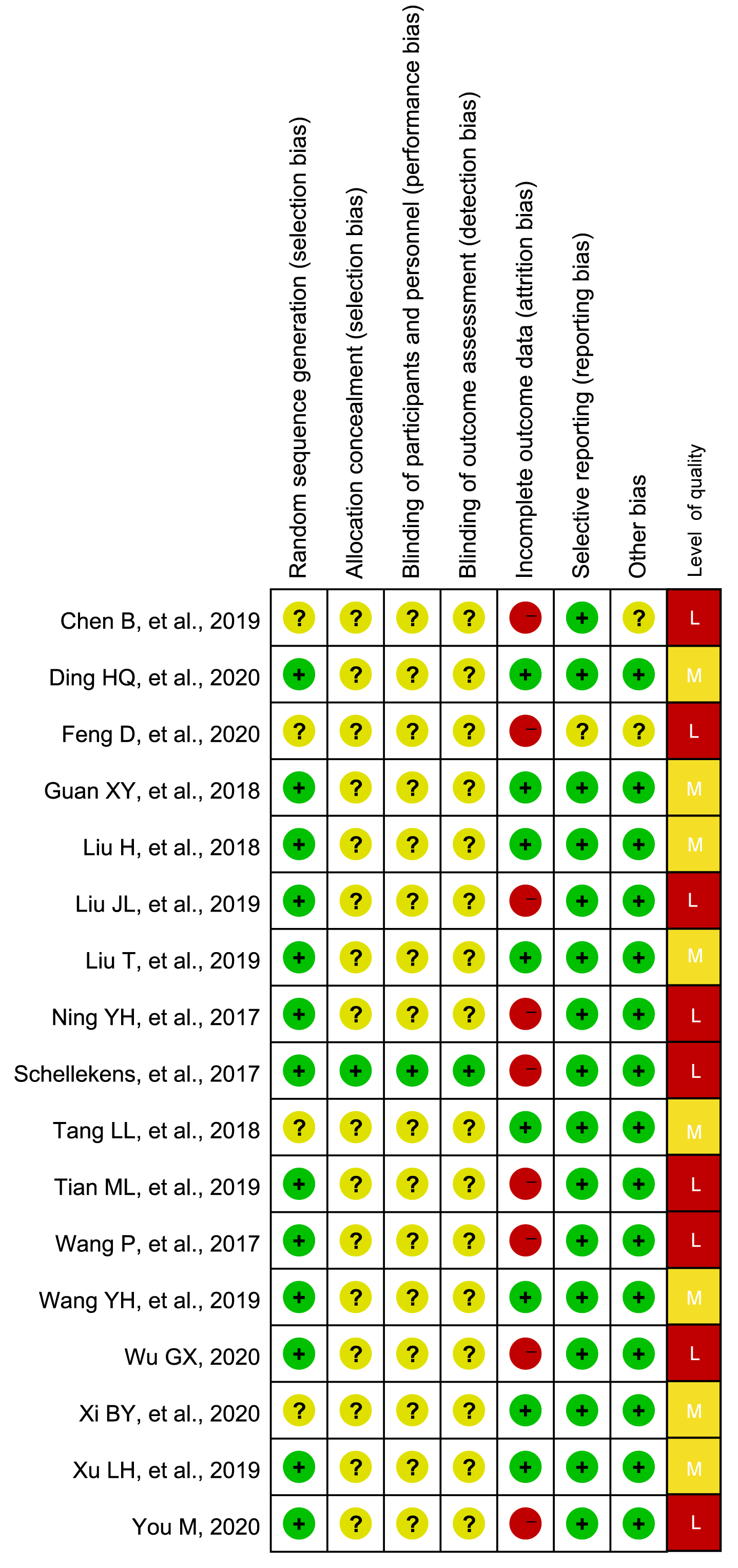

Supplement: Supplementary Figure 1 — Risk of bias of each eligible study. Green (+), yellow (?), and red (–) circle represents “low,” “unclear,” and “high” risk of bias, respectively. [file Image_1.TIF]
