# Supplementary material for: The Impact of Mindfulness-Based Stress Reduction (MBSR) on Psychological Outcomes and Quality of Life in Patients With Lung Cancer: A Meta-Analysis
Source: Front Psychol. 2022 Jun 28;13:901247. doi: 10.3389/fpsyg.2022.901247 (PMC9274275; doi:10.3389/fpsyg.2022.901247)
Supplement: Supplementary Table 1 — Search strategies of targeted English databases. [file Table_1.DOCX]

Search strategy of PubMed

| NO. | Query | Results |
| --- | --- | --- |
| 5 | (("Lung Neoplasms"[Mesh]) OR (((((((Pulmonary Neoplasms[Title/Abstract]) OR (Lung Neoplasm[Title/Abstract])) OR (Pulmonary Neoplasm[Title/Abstract])) OR (Lung Cancer[Title/Abstract])) OR (Lung Cancers[Title/Abstract])) OR (Pulmonary Cancer[Title/Abstract])) OR (Pulmonary Cancers[Title/Abstract]))) AND (((mindfulness-based stress reduction[Title/Abstract]) OR (mindfulness based stress reduction[Title/Abstract])) OR (MBSR[Title/Abstract])) | 8 |
| 4 | ((mindfulness-based stress reduction[Title/Abstract]) OR (mindfulness based stress reduction[Title/Abstract])) OR (MBSR[Title/Abstract]) | 1,207 |
| 3 | ("Lung Neoplasms"[Mesh]) OR (((((((Pulmonary Neoplasms[Title/Abstract]) OR (Lung Neoplasm[Title/Abstract])) OR (Pulmonary Neoplasm[Title/Abstract])) OR (Lung Cancer[Title/Abstract])) OR (Lung Cancers[Title/Abstract])) OR (Pulmonary Cancer[Title/Abstract])) OR (Pulmonary Cancers[Title/Abstract])) | 310,597 |
| 2 | ((((((Pulmonary Neoplasms[Title/Abstract]) OR (Lung Neoplasm[Title/Abstract])) OR (Pulmonary Neoplasm[Title/Abstract])) OR (Lung Cancer[Title/Abstract])) OR (Lung Cancers[Title/Abstract])) OR (Pulmonary Cancer[Title/Abstract])) OR (Pulmonary Cancers[Title/Abstract]) | 183,379 |
| 1 | "Lung Neoplasms"[Mesh] | 252,693 |

Search strategy of EMBASE

| NO. | Queries | Results |
| --- | --- | --- |
| #6. | #5 AND [embase]/lim | 14 |
| #5. | #1 AND #4 | 16 |
| #4. | #2 OR #3 | 469,010 |
| #3. | 'lung cancer'/exp | 424,678 |
| #2. | 'pulmonary neoplasms':ti,ab,kw OR 'lung neoplasm':ti,ab,kw OR 'pulmonary neoplasm':ti,ab,kw OR 'lung cancer':ti,ab,kw OR 'lung cancers':ti,ab,kw OR 'pulmonary cancer':ti,ab,kw OR 'pulmonary cancers':ti,ab,kw | 282,061 |
| #1. | 'mindfulness-based stress reduction':ti,ab,kw OR 'mindfulness based stress reduction':ti,ab,kw OR mbsr:ti,ab,kw | 1,799 |

Search strategy of the Cochrane library

| ID | Search | Results |
| --- | --- | --- |
| #1 | (Pulmonary Cancers):ti,ab,kw OR (Pulmonary Cancer):ti,ab,kw OR (Lung Cancers):ti,ab,kw OR (Lung Cancer):ti,ab,kw OR (Pulmonary Neoplasm):ti,ab,kw | 27948 |
| #2 | (Lung Neoplasm):ti,ab,kw OR (Pulmonary Neoplasms):ti,ab,kw | 4895 |
| #3 | #1 or #2 | 28661 |
| #4 | MeSH descriptor: [Lung Neoplasms] explode all trees | 8282 |
| #5 | #3 or #4 | 29528 |
| #6 | (mindfulness-based stress reduction):ti,ab,kw OR (mindfulness based stress reduction):ti,ab,kw OR (MBSR):ti,ab,kw | 1429 |
| #7 | #5 and #6 | 10 |

Search strategy of PsycINFO

| NO. | Queries | Results |
| --- | --- | --- |
| #1 | (Pulmonary Neoplasms or Lung Neoplasm or Pulmonary Neoplasm or Lung Cancer or Lung Cancers or Pulmonary Cancer or Pulmonary Cancers).tw. | 2811 |
| #2 | (mindfulness-based stress reduction or mindfulness-based stress reduction or MBSR).tw. | 1275 |
| #3 | 1 and 2 | 4 |

Search strategy of CNKI

检索范围 [Search scope]: (主题 [subject retrieval]: 正念减压 [mindfulness-based stress reduction/mindfulness decompression] (精确 [precise retrieval]) OR (主题: [subject retrieval]: 正念减压疗法 [mindfulness-based stress reduction (精确 [precise retrieval]) OR (主题 [subject retrieval]: MBSR (精确 [precise retrieval]) OR (主题 [subject retrieval]: 正念 [mindfulness] (精确 [precise retrieval]) AND ((主题 [subject retrieval]: 肺癌 [lung cancer] (精确 [precise retrieval]) OR (主题 [subject retrieval]: 肺瘤 [lung tumor] (精确 [precise retrieval]) OR (主题 [subject retrieval]: 肺肿瘤 (lung neoplasm (精确 [precise retrieval]) OR (主题 [subject retrieval]: 肺肿物 [lung carcinoma/mass] (精确 [precise retrieval])) 35

Search strategy of Wanfang Data

检索表达式 [search string] (主题词扩展 [subject extension retrieval]): 题名或关键词 [title/keywords]: (肺癌 [lung cancer] or 肺瘤 [lung tumor] or 肺肿瘤 [lung neoplasm] or 肺肿物 [lung carcinoma/mass]) and 题名或关键词 [title/keywords] :(正念 [mindfulness] or 正念减压 [mindfulness-based stress reduction/mindfulness decompression] or 正念减压疗法 [mindfulness-based stress reduction]) 52
